# Primary Localized Cutaneous Amyloidosis in Central Europe: A Retrospective Monocentric Study on Epidemiology and Therapy

**DOI:** 10.3390/jcm12247672

**Published:** 2023-12-14

**Authors:** Sára Pálla, Enikő Kuroli, Eszter Alexa Tóth, Bernadett Hidvégi, Péter Holló, Márta Medvecz

**Affiliations:** 1 Department of Dermatology, Venereology and Dermatooncology, Semmelweis University, 1085 Budapest, Hungary; 2 Department of Pathology and Experimental Cancer Research, Semmelweis University, 1085 Budapest, Hungary

**Keywords:** amyloidosis, epidemiology, therapeutics, dermoscopy, histology, Thioflavin T, comorbidity, macular amyloidosis, lichen amyloidosis, atopic dermatitis, pruritus

## Abstract

Amyloid deposits can be the cause of many chronic diseases. Primary localized cutaneous amyloidosis (PLCA) is a chronic dermatologic condition with amyloid deposits in the papillary dermis. The most common types of the keratinocyte-derived form of PLCA include macular (MA), lichen (LA), and biphasic (BA) amyloidosis. The estimated prevalence of PLCA in the Asian population is 0.98/10,000, which is higher than in the European population; thus, epidemiologic data on PLCA in the Caucasian population are limited. We performed a retrospective single-center study analyzing epidemiologic characteristics of a Central European PLCA population. Epidemiologic data regarding age, sex, skin phototype (Fitzpatrick scale I–VI), disease duration, comorbidities, history of atopy, and family history of PLCA were collected. Clinical characteristics, localization of PLCA lesions, applied therapies and treatment outcomes were also analyzed. Dermoscopic characteristics were also evaluated. A total of 41 patients diagnosed with PLCA were included, with 22 presenting with macular, 18 with lichen, and 1 with biphasic amyloidosis. The male/female ratio was 16/25, and mean age at diagnosis was 54.6 ± 15.2 years (range 27–87 years). The mean age at the onset of PLCA was 53 ± 16.1 years (range 19–79 years) in MA, 46.7 ± 18.2 years (range 14–73 years) in LA, and 26 years in BA. The interscapular region in MA and the extensor surface of the lower extremities in LA proved to be localization-related areas. In our center, a wide range of therapeutic options was applied, with the most prescribed being topical corticosteroids in all types of PLCA. We presented a retrospective, monocentric study on the epidemiology of PLCA in the Central European region. By examining the medical data of a significant number of PLCA patients, we compared our epidemiologic data with that of the Asian PLCA population. Due to the rarity of the condition, further randomized controlled trials and guidelines are needed to improve therapeutic outcomes.

## 1. Introduction

Primary localized cutaneous amyloidosis (PLCA) is a rare, chronic dermatologic disease characterized by the deposition of heterogenous amyloid proteins in the papillary dermis of the skin without systemic involvement. The disease is more common in the Asian population, where the estimated prevalence is 0.98/10,000 [1,2]. Epidemiologic data describing the Caucasian population are therefore immensely limited [3,4].

There are three subtypes of primary localized cutaneous amyloidosis: macular amyloidosis (MA), lichen amyloidosis (LA), and nodular amyloidosis (NA) [5,6]. Biphasic amyloidosis (BA) is the combined appearance of MA and LA. MA, LA, and BA are keratinocyte-derived, in which cytokeratins serve as amyloid precursors; while NA is immunoglobulin light-chain-derived and is associated with dermal plasma cell infiltration. In this study, our focus is on the keratinocyte-derived forms of PLCA.

MA is characterized by a pruritic, hyperpigmented macule of variable extent, and commonly presents as having a “rippled” or reticulate appearance. MA is often accompanied by pruritus, but asymptomatic cases have also been described. LA is the most common form of PLCA, usually presenting as strongly pruritic, grouped, skin-colored or hyperpigmented, dome-shaped or hemispheric papules that later coalesce into plaques. LA is commonly located on the extensor surfaces of the extremities [7]. In BA, hyperpigmented macules and grouped papules are both present. Patients diagnosed with PLCA experience a significant impairment in Quality of Life (QoL), with their mean DLQI score measured as 9.05 in a previous study [8]. In a recent study, MA was associated with diminished QoL, that are influenced by factors such as age, location of skin lesions (covered or uncovered), and severity of pruritus [9].

Dermoscopic characteristics of MA and LA have been described in a few studies, with a recent cross-sectional investigation including an Asian PLCA population [10]. In the majority of the cases, dermoscopic images of LA showed central white hubs or scar-like centers surrounded by grey–brown dots (<0.1 mm), globules (>0.1 mm), or peppering (the accumulation of multiple very small (<0.1 mm) blue–grey dots) [11]. Previous reports used the term ‘two-zone pattern’ to describe this arrangement of dermoscopic structures [12]. Other findings in LA included blue–grey ovoid nest-like areas, white scaling, shiny white streaks under polarized light, white collarettes, and concentric structures [13]. In LA, different vascular structures (branching, dotted and globular vessels) in between the white hubs were also observed [12].

Dermoscopic characteristics of MA have been described as white, brown, or mixed central hubs. The peripheral pigmentation was observed as fine streaks, leaf-like extensions, or bulbous projections, while others reported semicircular hyperpigmented structures [13]. Pigment dots, globules or peppering has also been noted [11]. Previous reports described the arrangement of the structures using the term ‘jigsaw puzzle pattern’ [12].

The pathogenesis of PLCA is not fully understood; it is considered to be multifactorial, involving both environmental and genetic factors. Immunohistochemical studies have suggested that the major constituents of amyloid deposits are keratins, galectin-7 and actin [14]. Keratin 5/14 (K5/14) is a heterodimeric protein composed of keratin 5 (K5) and keratin 14 (K14). It is the major component of keratinocytes in the basal cell layer of the epidermis. Fibulin-4 is an elastin-associated protein and thus a constituent of the elastic fibers in the dermis. A recent study has shown that K5 degradation fragments form amyloid fibrils with fibulin-4 and potentially promote cutaneous amyloidosis [15].

Up to 10% of all PLCA cases are familial, where an autosomal dominant inheritance can be observed [16,17]. In cases of familial PLCA, pathogenic heterozygous missense mutations have been mapped to the *OSMR* gene encoding the interleukin-31 (IL-31) receptor subunit oncostatin-M receptor ß (OSMRß), and the *IL31RA* gene encoding the IL-31 receptor alpha, which pairs with OSMRß to form the IL-31 receptor [18,19,20,21]. These receptors are members of the IL-6 cytokine receptor family, and work through the JAK/STAT, MAPK and PI3K/Akt signal transduction pathways. They play an important role through their cytokines (IL-6, IL-11, IL-27, IL-31 and oncostatin M) in keratinocyte differentiation, proliferation, apoptosis, and inflammation, that are pivotal in the pathogenesis of PLCA [22,23]. It has been suggested that pathogenic mutations in *OSMR* and *IL31RA* genes lead to incorrect IL-31 signaling, which is directly related to pruritus [24].

Most cases of PLCA are idiopathic; however, associations with pachyonychia congenita, familial palmoplantar keratoderma and multiple endocrine neoplasia type 2a (MEN2A) have been reported [7,25,26]. The strongest association has been defined with MEN2A, which is a rare autosomal dominant endocrine tumor syndrome, characterized by medullary thyroid carcinoma, pheochromocytoma and parathyroid tumors, caused by a heterozygous pathogenic mutation in the *RET* gene [26].

The diagnosis of PLCA should be confirmed by histopathology, where the deposition of eosinophil, amorphous globular material in the papillary dermis, melanophages, and increased pigmentation of the basal layer are typically observed. In LA, acanthosis and hyperkeratosis can also be seen. The reduced number of nerves within the dermo-epidermal junction and the epidermis has also been reported [27]. Special stains (Congo red, Crystal violet, Thioflavin T, periodic-acid-Schiff method, Sirius red) can be used for more defined visualization of the amyloid [7,28].

To date, many topical, systemic agents, phototherapy, and laser therapeutic modalities for PLCA have been described in numerous case reports. However, only a few clinical trials have been performed, and there are no therapeutic guidelines available. Systemic retinoid treatment in LA and BA decreased pruritus and the size of the papules, while topical retinoid was reported to result in mixed outcomes in LA and MA [24,29]. Topical vitamin D_3_ analogue calcipotriol showed beneficial effects on the symptoms of LA [30]. Oral application of cyclophosphamide decreased pruritus, hyperpigmentation, and the size of the lesions [31]. Topical corticosteroids or dimethyl sulfoxide (DMSO) have been used with varying degrees of success [24]. Topical calcineurin-inhibitors cyclosporine and tacrolimus have been successfully used in LA [31,32,33]. Colchicine showed beneficial effects on pruritus, hyperpigmentation, and the size of the papules in a single publication [34]. The topical application of capsaicin resulted in clinical and histological improvement of LA lesions but showed no beneficial effects in MA [34,35]. Additionally, topical application of menthol has been reported with satisfactory outcomes [36], and methotrexate was effective in resolving skin manifestations and reducing pruritus in LA patients [37].

Many publications have shown the efficiency of laser therapy on PLCA [38]. Carbon dioxide laser has demonstrated successful clinical outcomes in the treatment of MA and LA lesions [39,40]. Decreased pruritus was reported after treatment with ytterbium/erbium laser [41], and the application of pulsed dye laser has been recognized as beneficial in MA and LA [42,43]. Erbium: yttrium aluminum garnet laser and neodymium-doped: yttrium aluminum garnet laser therapies have been efficient in MA and LA patients as well [44,45,46,47].

Different phototherapeutic modalities have also been described in the treatment of PLCA. While narrow-band UVB irradiation has reportedly shown favorable results on patients with LA, BA and MA [48,49,50], topical psoralen with ultraviolet A (PUVA) therapy has been used with mixed outcomes in a few studies [51,52]. Furthermore, additional interventions such as transcutaneous nerve stimulation in MA, and surgical procedures (electrodessication, dermabrasion) in LA have also been reported [24].

Data on PLCA in the Caucasian population are limited due to its low prevalence. The aim of this retrospective study was to analyze the epidemiology of PLCA in a single center in the Central European region and compare our findings with the data assessed in the Asian PLCA population. We also analyzed various treatment regimens for its management in our center and determined the response to therapy in each PLCA case. Through this study, our goal is to expand our knowledge of this rare cutaneous disease and share our clinical data, dermoscopic images, and clinical experience with associated therapeutic possibilities.

## 2. Materials and Methods

### 2.1. Patients

In this retrospective study, we retrieved the data of cases encoded as PLCA by the International Statistical Classification of Diseases and Related Health problems code of amyloidosis from the medical data recording and text retrieval system at our department between 1 January 2004 and 1 September 2022. The study was reviewed and approved by the Semmelweis University Regional and Institutional Committee of Science and Research Ethics (license number: 169/2022.). The diagnosis of PLCA was established by clinical examination and proven by histopathological examination of skin biopsy specimens from affected skin sites. All specimens were reviewed by an expert dermatopathologist. In addition to hematoxylin and eosin (H&E) staining, we performed Thioflavin T staining, which is selective for amyloid fibrils, in all cases.

### 2.2. Dermoscopic Imaging

Dermoscopic images of selected lesions were evaluated for the presence of specific features. Vascular structures were also analyzed. The dermoscopic images were obtained using an Illuco IDS-1100 dermatoscope (Illuco, Gunpo, Republic of Korea). In this work, we used the standard dermoscopic terminology as established by Errichetti et al. [53].

### 2.3. Clinical Data Evaluation

We classified PLCA subtypes as lichen, macular and biphasic based on medical data, histological findings and clinical photodocumentation, by analyzing both specific and non-specific features. The distribution of PLCA symptoms was determined and divided according to the following body areas: head, back (differentiating scapular and interscapular; thoracic, and lumbar regions), chest, abdomen, and upper and lower extremities (differentiating shoulders, gluteal region, extensor surface, flexor surface, hands, and feet). Age, sex, skin phototype (Fitzpatrick scale I–VI), disease duration, comorbidities, history of atopy, and family history of PLCA were also analyzed. Treatment modalities were identified and response to therapy was classified according to the documented changes in the symptoms (worsening symptoms, unchanged symptoms, decreasing pruritus, and objective improvement assessed by the clinician). Potency of the applied topical corticosteroids (TCSs) was categorized according to the Anatomical Therapeutic Classification (ATC) of the World Health Organization (Geneva, Switzerland) [54].

### 2.4. Statistical Analysis

SPSS Statistics version 27 (IBM, Armonk, NY, USA) was used to perform statistical analysis. Results are expressed as mean ± SD. Continuous variables were compared using Mann–Whitney U-test and dichotomous variables were compared using chi-squared test. A *p*-value of <0.05 was taken to be statistically significant.

## 3. Results

**Figure 1:**
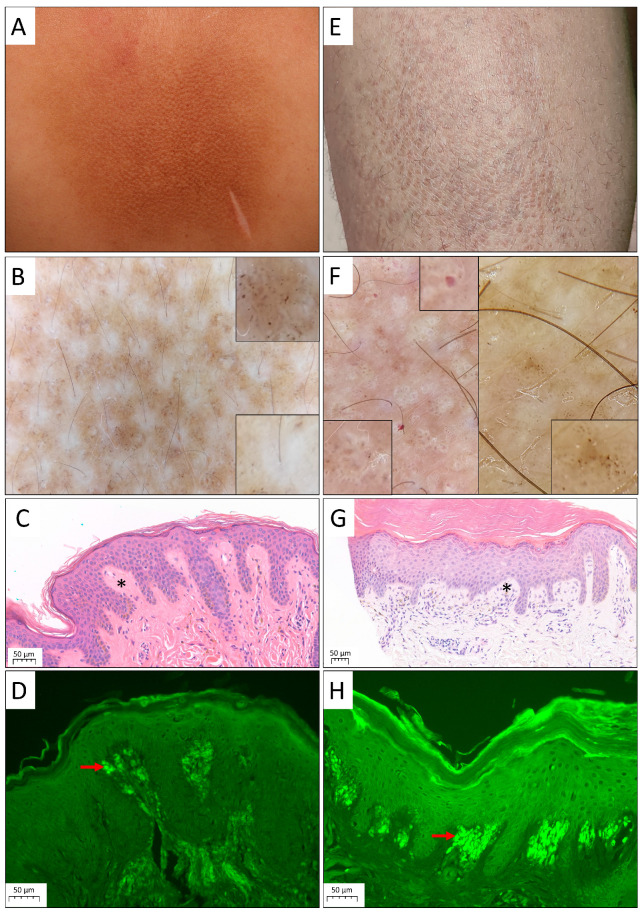
Clinical and histological characteristics of macular (MA) and lichen amyloidosis (LA) types of primary localized cutaneous amyloidosis. (**A**) Macular amyloidosis, hyperpigmented macule on the back. (**B**) Dermoscopy of MA, ×10. White central structureless areas surrounded by brown and grey dots. (**C**) Histology of MA, revealing amyloid deposits presented as eosinophil globular material in the papillary dermis (asterisk), H&E. (**D**) Thioflavin T staining of MA. Red arrow indicates amyloid deposits. (**E**) Lichen amyloidosis, small papules on the shin. (**F**) Dermoscopy of LA, ×10. Brown globules are surrounded by white circular structureless areas. Dotted vessels, and white structureless areas surrounded by brown and grey dots are also present. (**G**) Histology of LA, showing amyloid deposits presented as eosinophil globular material in the papillary dermis (asterisk), H&E. (**H**) Thioflavin T staining of LA. Red arrow indicates amyloid deposits.

A total of 41 patients diagnosed with PLCA (sex ratio 16/25; male/female) were included in our study, mean age at diagnosis was 54.6 ± 15.2 years (range 27–87 years). Diagnosis was confirmed by the histological examination of a skin biopsy specimen in all cases. Subsequently, 22 patients (53.7%) presented with MA, 18 patients (43.9%) with LA, and 1 (2.4%) with BA. There was no NA case diagnosed in our department during the examined time frame. Examples of clinical manifestation, histopathologic findings, and dermoscopic images of PLCA are shown in Figure 1. Mean age at onset of PLCA was 53 ± 16.1 years (range 19–79 years) in MA, 46.7 ± 18.2 years (range 14–73 years) in LA, and 26 years in BA. Mean time to diagnosis was 3.1 ± 2.4 years (range 0–8 years) in MA and 7.8 ± 6.9 years (range 0–25 years) in LA, time to diagnosis in the BA case was 5 years.

### 3.1. Sex Differences

In our study group, mean age at onset was lower for females than males (44.9 ± 15.7 vs. 55.5 ± 19.6; females vs. males). As for the MA:LA ratio, females were more likely to suffer from MA than males (16:8 (2) vs. 6:10 (0.6), females vs. males). However, none of these differences were statistically significant.

### 3.2. Skin Phototype (Fitzpatrick Skin Scale)

The patients’ skin phototype was determined according to the Fitzpatrick scale, based on the clinical photodocumentation. The skin of 26 patients was classified as phototype III (63.4%), and the skin of 3 other patients as phototype IV (7.3%). Other skin phototypes were not represented in our study. We were unable to determine the skin phototype of 12 patients (29.3%) due to the lack of clinical documentation.

### 3.3. Distribution of the Lesions

**Figure 2:**
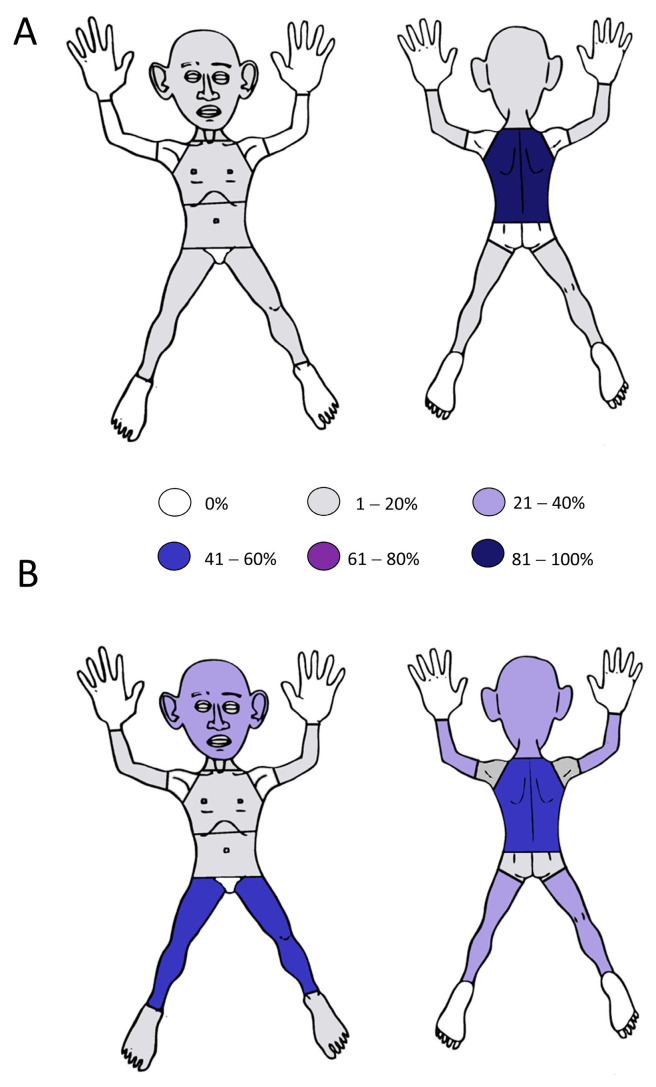
Distribution of the involved body regions in macular (**A**) and in lichen (**B**) amyloidosis types of primary localized cutaneous amyloidosis.

MA lesions were commonly located on the back (19 patients, 86.4%), especially in the scapular and interscapular region (11 patients, 50%) followed by the abdomen and the legs (3 patients each, 13.6%). Meanwhile, 3 patients had multiple sites of involvement (Figure 2). In LA, the most commonly affected regions were the lower extremities (11 patients, 61.1%), especially the extensor surface of the lower limbs (10 patients, 55.6%), followed by the back (9 patients, 50%); 7 patients had multiple sites involved. The data on all involved regions are included in Table 1. The one case of biphasic amyloidosis affected the patient’s head, back (scapular, thoracic and lumbar regions), flexor surfaces of upper extremities, and extensor surfaces of lower extremities. Pruritus was present in 18 (81.8%) and 17 cases (94.4%) in MA and in LA respectively, and in the 1 case of BA as well (85.4% of all PLCA cases).

### 3.4. Comorbidities

Chronic coexisting conditions were also evaluated. The main chronic conditions that patients suffered from were endocrine and metabolic disorders, including dyslipidemia, thyroid disorder, diabetes mellitus or other adrenal and parathyroid diseases, one patient had multiple endocrine neoplasia type 2A (MEN2A). A large number of the patients had cardiovascular disorder, including hypertension. Atopy was present in five patients; two patients were diagnosed with atopic dermatitis. There were no significant differences in the distribution of the comorbidities among the subtypes. Other associated comorbidities are included in Table 2. Family history for PLCA was positive in one case. In 21 patients (51.2%), serum protein electrophoresis was also performed to rule out systemic amyloidosis.

### 3.5. Treatment

Patients received either topical treatment, phototherapy, systemic treatment, or combination therapy; in seven cases, patients were recommended to use emollient without active ingredients. In our study, in both forms of amyloidosis, the most frequently applied therapy was TCS (13 MA and 15 LA patients). In LA, group III and IV corticosteroids were most commonly used (*n* = 14, 77.8%); and in eight cases, an objective improvement in symptoms was observed. In MA, group III and IV corticosteroids improved symptoms in four cases. Altogether, TCSs improved the symptoms of PLCA in 13 out of 28 patients (46% of patients treated with PLCA). Topical calcineurin inhibitor was applied in two MA cases and one LA case. Other treatment modalities and their effect on the symptoms are summarized in Table 3. In BA, the patient was treated with oral retinoid, and an objective improvement in symptoms was noted.

### 3.6. Dermoscopic Characteristics

Specific features and vascular structures were observed upon dermoscopic evaluation of three MA patients and one with LA. The dermoscopic examination of MA showed white central structureless areas around the follicles, surrounded with brown and grey dots (Figure 1B). In LA, structureless white areas were observed in a more irregular pattern, surrounded by brown and grey dots and globules. In other areas, brown globules were observed, surrounded by white circular structureless areas. Dotted vessels were also noticed in the lesions, in an unspecific arrangement (Figure 1F).

## 4. Discussion

The aim of this monocentric retrospective study was to analyze primary localized cutaneous amyloidosis in the Central European region concerning demographic data, body distribution, comorbidities, treatment practices and treatment outcomes. While PLCA is more prevalent in Asian and South American populations, epidemiologic data regarding PLCA in the Caucasian population are limited [2,3,55,56,57].

Multiple investigations have been conducted previously on the commonly involved body regions in Asian and South American populations [7,58,59,60]. The predilection sites for PLCA subtypes in a Caucasian population in our study were consistent with previous data from Asian populations: the interscapular region in MA and the extensor surface of the lower extremities in LA were identified as localization-related areas.

In a previous study on PLCA patients by Bandhlish et al. from Northern India, the mean age of onset for MA was measured as 34.6 ± 10.5 years [58]. In another study on the sex differences of Asian PLCA patients conducted by Lu et al., females were found to develop PLCA earlier (median age of onset was 28 in females, and 32 in males) and were more likely to suffer from MA than males (MA:LA ratio was 0.16 for males and 0.36 for females) [57]. Similar sex differences were found in our study; however, in our PLCA population, the MA:LA ratio was higher in both female and male patients. In our patient cohort, the onset age of PLCA was notably higher when compared to Asian patient populations.

In a recent retrospective monocentric study investigating the epidemiologic characteristics of PLCA in a Swiss center, females were found to be more likely to develop PLCA, and LA was the most frequent subtype [3]. Our study also included a higher proportion of female patients; however, MA was more common.

Diagnosing PLCA can be challenging due to its non-specific clinical manifestations, and the time to diagnosis could take many years. The differential diagnoses for MA include post-inflammatory hyperpigmentation, erythema dyschromicum perstans, drug-induced pigmentation, frictional melanosis, and notalgia paraesthetica. LA should be differentiated from lichen planus, chronic lichen simplex, prurigo simplex, and prurigo nodularis [7,61]. The role of histology and specific stains for amyloid deposits, such as Congo red and Thioflavin T, is significant, as none of these other entities exhibit amyloid deposition.

Previous reports on the dermoscopic features of PLCA were mostly presented using subjective metaphoric language [12]. Here, we provided descriptions of previously reported terms, such as ‘two-zone pattern’ and ‘jigsaw puzzle pattern’, using standard dermoscopic terminology.

A strong association between PLCA and atopy was observed in a population-based study from Taiwan; 7% of the study cohort with PLCA was diagnosed with atopic dermatitis (AD), and 2.7% with AD along with asthma and/or allergic rhinitis [1]. Similarly, a strong association between PLCA and AD has been established among Asian patients [19]. Hyperlipidemia and diabetes mellitus were significantly associated with PLCA in the Taiwanese population [1]. We observed dyslipidemia in 22% and AD in 4.9% of our patients diagnosed with PLCA. Diabetes mellitus, AD, and thyroid disease often lead to chronic pruritus and scratching, contributing to epidermal damage that can promote the formation of amyloid deposits. While these conditions may facilitate the development of PLCA, it is considered to be a multifactorial disease requiring additional contributing factors.

Pruritus is a common finding in PLCA; however, previous studies report its absence in 10–40% of all PLCA patients [59,62,63]. In our study, pruritus was absent in 12.2% of all PLCA cases (18.2% of MA and 5.6% of LA cases). Recently, generalized, non-pruritic LA has also been reported in the literature in a few cases [64,65]. These data indicate that while pruritus can contribute to the development of the disease, not all PLCA cases arise due to chronic scratching.

The association between MEN2A and LA has been reported previously, defining it as the second most common manifestation of MEN2A [66]. Our patient with MEN2A-related PLCA carries a mutation in codon 634 of the *RET* gene. This genetic alteration causing MEN2A was previously linked with PLCA [26]. Despite the potential genetic background, histopathology showed no difference between MEN2A-associated PLCA and PLCA without MEN2A in previous studies [66]. In our patient with MEN2A, the clinical picture and the histopathology of LA did not differ from other LA cases.

There is a lack of supporting evidence for the systemic involvement of MA or LA [7]. A rare atypical bullous variant of LA has been described in some case reports, where later systemic amyloidosis was also diagnosed. Therefore, attention should be given to PLCA patients presenting with pruritic lichenoid papules and vesicles [67].

In this study, we examined the therapy in a large PLCA patient population. There are no guidelines for an evidence-based therapy regimen for PLCA, and a wide range of treatment options are reported in the literature [24]. Topical corticosteroids (TCSs) are the gold-standard treatment for numerous inflammatory skin diseases; however, the adverse effects of long-term TCSs should always be considered [68]. According to previous studies, the long-term effect of TCSs on PLCA is debatable [30,69]. In our PLCA patient cohort, symptomatic improvement with TCSs was observed in 46% of patients treated with them. A total of eight patients were lost to follow-up; however, complete remission of symptoms or well-controlled disease could also be suspected in these cases. The application of topical calcineurin inhibitors have been reported to have beneficial effects on PLCA symptoms [31]. In our study, objective improvement in symptoms was reported in both MA cases where topical calcineurin inhibitor was used.

It has been previously reported that dupilumab can be an effective treatment for pruritus in AD patients [70]. By blocking IL-4 and IL-13 on sensory neurons and inhibiting IL-31 production, it may decrease pruritus in PLCA patients as well. Dupilumab has resolved pruritus and decreased the size of the lesions, thus improving the quality of life for LA patients with generalized symptoms, as described in recent case reports [71,72]. However, further studies are necessary to determine the long-term efficacy and safety of this new therapeutic option among PLCA patients.

## 5. Conclusions

We conducted a retrospective, monocentric study on the epidemiology of PLCA in the Central European region. By examining the medical data of a significant number of PLCA patients, we compared our epidemiologic data with those of the Asian PLCA population, identifying differences in the age of onset of PLCA. The treatment regimens used in our center were also analyzed. Corticosteroids were the most frequently used treatment; however, the long-term efficacy regarding the symptomatic relief remains questionable. Given the rarity of this chronic cutaneous disease, further randomized controlled trials on newly available treatment options are needed to improve therapeutic possibilities. Further, guidelines for an evidence-based therapeutic regimen are also necessary.

## Figures and Tables

**Table 1:** Involved regions in macular amyloidosis (*n* = 22), in lichen amyloidosis (*n* = 18), and in all primary localized cutaneous amyloidosis (*n* = 41) cases.

Involved Regions		Macular Amyloidosis *n* (% of MA Cases)	Lichen Amyloidosis *n* (% of LA Cases)	All PLCA Cases *n* (% of All Cases)
Head		1 (4.5)	4 (22.2)	6 (14.6)
Back		19 (86.4)	9 (50)	29 (70.7)
	Scapular and interscapular	11 (50)	5 (27.8)	17 (41.5)
	Thoracic	7 (31.8)	1 (5.6)	9 (22)
	Lumbar	3 (13.6)	3 (16.7)	7 (17.1)
Chest		2 (9.1)	2 (11.1)	4 (9.8)
Abdomen		3 (13.6)	3 (16.7)	6 (14.6)
Upper extremities		1 (4.5)	7 (38.9)	9 (22)
	Shoulders	-	2 (11.1)	2 (4.9)
	Flexor surface	-	1 (5.6)	2 (4.9)
	Extensor surface	1 (4.5)	5 (27.8)	6 (14.6)
Lower extremities		3 (13.6)	11 (61.1)	15 (36.6)
	Gluteal	-	2 (11.1)	2 (4.9)
	Flexor surface	2 (9.1)	4 (22.2)	6 (14.6)
	Extensor surface	1 (4.5)	10 (55.6)	12 (29.3)
	Feet	-	1 (5.6)	1 (2.4)

LA: lichen amyloidosis, MA: macular amyloidosis, PLCA: primary localized cutaneous amyloidosis.

**Table 2:** Comorbidities of patients diagnosed with primary localized cutaneous amyloidosis.

Comorbidities		Number of Cases (%)
Cardiovascular		14 (34.1)
	Hypertension	12 (29.3)
	Other (Coronary artery disease, stroke, atrial fibrillation)	4 (9.8)
Musculoskeletal (Rheumatoid arthritis)		2 (4.9)
Urogenital (Prostate hyperplasia and myoma)		3 (7.3)
Digestive (GERD, diverticulosis, and viral hepatitis)		4 (9.8)
Endocrine and metabolic		17 (41.5)
	Dyslipidemia	9 (22)
	Thyroid disorder	5 (12.2)
	Diabetes	3 (7.3)
	Other (adrenal and parathyroid disorder)	2 (4.9)
Neurological and psychiatric (migraine, depression, affective disorder, and anxiety disorder)		4 (9.8)
Malignancies		5 (12.2)
Atopy (Atopic dermatitis, allergic rhinitis, or asthma)		5 (12.2)

GERD: gastroesophageal reflux disease.

**Table 3:** Treatment modalities and treatment efficacy in different subtypes of primary localized cutaneous amyloidosis.

Treatment	MA		LA		All Patients, *n* (%)
	n	Effect on Symptoms	n	Effect on Symptoms	
Topical therapy					
Weak and moderately potent CSs (groups I and II)	2	P (1), U (1)	1	U (1)	3 (7.3)
Potent and very potent CSs (groups III and IV)	11	P (1), O (3), U (5), L (2)	14	U (2), O (8), L (4)	25 (61)
Capsaicin cream	2	L (2)	1	P (1)	3 (7.3)
Calcineurin inhibitor	2	O (2)	1	L (1)	3 (7.3)
Phototherapy					
PUVA	-	-	3	U (1), W (2)	3 (7.3)
nb-UVB	-	-	1	U (1)	1 (2.4)
Systemic treatment					
Oral retinoid	-	-	1	U (1)	2 (4.9)
Combination treatment					
nb-UVB and very potent TCS	1	O (1)	1	O (1)	2 (4.9)
Oral retinoid and very potent TCS	-	-	1	U (1)	1 (2.4)
Bland emollient	6		1		7 (17.1)

CSs: corticosteroids, L: lost to follow-up, LA: lichen amyloidosis, nb-UVB: narrowband ultraviolet B, MA: macular amyloidosis, O: objective improvement, P: pruritus relieved, PUVA: psoralen plus ultraviolet A, TCS: topical corticosteroid, U: unchanged symptoms, W: worsening symptoms.

## Data Availability

The datasets analysed during the current study are available from the corresponding author upon reasonable request.
